# Model-informed precision dosing of quetiapine in bipolar affective disorder patients: initial dose recommendation

**DOI:** 10.3389/fpsyt.2024.1497119

**Published:** 2024-12-04

**Authors:** Zi-Qiang Zheng, Ying-Wei Jin, Di Yin, Xiao Chen, Su-Mei He, Chen-Xu Liu, Cun Zhang, Dong-Dong Wang

**Affiliations:** ^1^ Department of Pharmacy, The Affiliated Lianyungang Hospital of Xuzhou Medical University, Lianyungang, Jiangsu, China; ^2^ Jiangsu Key Laboratory of New Drug Research and Clinical Pharmacy & School of Pharmacy, Xuzhou Medical University, Xuzhou, Jiangsu, China; ^3^ Department of Pharmacy, The Suqian Clinical College of Xuzhou Medical University, Suqian, Jiangsu, China; ^4^ Department of Pharmacy, Wuxi Maternity and Child Health Care Hospital, Wuxi, Jiangsu, China; ^5^ School of Nursing, Xuzhou Medical University, Xuzhou, Jiangsu, China; ^6^ Department of Pharmacy, Suzhou Research Center of Medical School, Suzhou Hospital, Affiliated Hospital of Medical School, Nanjing University, Suzhou, Jiangsu, China; ^7^ Department of Pharmacy, Shenzhen Hospital, Southern Medical University, Shenzhen, China; ^8^ Department of Pharmacy, Xuzhou Oriental Hospital Affiliated to Xuzhou Medical University, Xuzhou, Jiangsu, China

**Keywords:** model-informed precision dosing, quetiapine, bipolar affective disorder, initial dose recommendation, real-world data

## Abstract

**Objective:**

Bipolar affective disorder (BAD) is a mood disorder with high morbidity and mortality. Quetiapine can be used in the treatment of patients with BAD; however, the precise administration regimen of quetiapine in these patients is still unknown. In this study, a population pharmacokinetic (PPK) model of quetiapine in patients with BAD was constructed based on model-informed precision dosing (MIPD) and real-world clinical data and an optimal initial dose of quetiapine in these patients was recommended.

**Methods:**

A total of 99 patients with BAD treated with quetiapine were included. At the same time, the quetiapine concentrations, the physical and chemical indices of the patients, and the drug combination information were collected. A quetiapine PPK model for patients with BAD was then constructed and an initial dose based on Monte Carlo simulation was recommended.

**Results:**

In the final model of quetiapine for patients with BAD, the apparent oral clearance (CL/F) and the apparent volume of distribution (V/F) were 76.1 L/h and 530 L, respectively. For patients with BAD weighing 40–66 kg, the initial dose recommendation was 16 mg kg^−1^ day^−1^, the probability of reaching the therapeutic window was 78.8%–82.2%, and the probability of exceeding the upper limit of the therapeutic window was 5.2%–10.3%. For patients with BAD weighing 66–120 kg, the initial dose recommendation was 12 mg kg^−1^ day^−1^, the probability of reaching the therapeutic window was 81.5%–85.5%, and the probability of exceeding the upper limit of the therapeutic window was 3.6%–8.1%.

**Conclusion:**

The present study, for the first time, recommended an initial dose of quetiapine in patients with BAD based on MIPD and real-world data, providing an individualized reference for the administration of quetiapine in these patients.

## 1 Introduction

Bipolar affective disorder (BAD) is a mood disorder that is clinically manifested as manic episodes or as both depressive and manic episodes (1–6). Studies found that anxiety disorders have become the first major category of mental diseases, followed by mood disorders, the second major category of mental diseases, with a prevalence rate of 0.8%–9.6%. The lifetime prevalence of BAD is 1.02% (7, 8). BAD has high disability and fatality rates, and its all-cause mortality rate is higher than that of the general population, approximately twice that of the general population. In addition, studies found that the suicide rates of patients with BAD are the highest among all mental diseases, with the suicide risk being 20–30 times higher than that of the general population (9–14). In short, the learning, work, and self-care abilities of patients with BAD are reduced and the quality of life is decreased, as well as even suicide in severe cases, causing serious trauma and economic burden to families and society.

Quetiapine is an atypical antipsychotic drug, which is an antagonist of various neurotransmitter receptors in the brain and can be applied for all types of schizophrenia (15–18). Furthermore, quetiapine can reduce the emotional symptoms associated with schizophrenia, such as depression, anxiety, and cognitive deficits, and can also be used in the treatment of BAD (19–22). However, the pharmacokinetic variation of quetiapine is large, and it is difficult to formulate the optimal drug administration regimen in clinical practice. In this study, a population pharmacokinetic (PPK) model of quetiapine in patients with BAD was built based on model-informed precision dosing (MIPD) and real-world clinical data and an optimal initial dose of quetiapine for these patients was recommended.

## 2 Methods

### 2.1 Data collection and patient information

Patients with BAD who were treated with quetiapine from Xuzhou Oriental Hospital affiliated with Xuzhou Medical University between July 2020 and August 2023 were included, retrospectively. The criteria for inclusion were as follows: i) diagnosed with BAD; ii) treated with quetiapine; and iii) therapeutic drug monitoring (TDM) for quetiapine. The research was approved by the Research Ethics Committee of Xuzhou Oriental Hospital affiliated with Xuzhou Medical University. The quetiapine concentrations in patients with BAD, along with the physiological and biochemical indicators, and the concomitant medications were collected from real-world data, including TDM and medical record system.

A total of 99 patients with BAD treated with quetiapine were included for analysis, which comprised 66 men and 33 women aged 17.05–69.47 years and weighing 43.00–119.00 kg. The demographic data for BAD and the drug combinations are shown in **Tables 1** and **2**, respectively.

**Table 1:** Demographic data of patients with bipolar affective disorder treated with quetiapine (*n* = 99).

Characteristics	Mean ± SD	Median (range)
Gender (men/women)	66/33	–
Age (years)	39.05 ± 12.79	36.66 (17.05–69.47)
Weight (kg)	73.75 ± 13.85	72.00 (43.00–119.00)
Albumin (g/L)	41.80 ± 3.49	41.65 (33.60–50.40)
Globulin (g/L)	27.31 ± 3.99	27.50 (18.90–41.30)
Alanine transaminase (IU/L)	29.01 ± 31.81	19.00 (7.00–308.00)
Aspartate transaminase (IU/L)	23.89 ± 18.66	19.00 (11.00–212.00)
Creatinine (μmol/L)	68.11 ± 17.88	68.00 (32.00–150.00)
Urea (mmol/L)	4.50 ± 1.76	4.20 (1.99–12.49)
Total protein (g/L)	69.12 ± 5.60	69.10 (56.80–84.90)
Total cholesterol (mmol/L)	4.62 ± 1.01	4.59 (2.45–7.53)
Triglyceride (mmol/L)	2.28 ± 1.12	2.14 (0.70–5.74)
Direct bilirubin (μmol/L)	2.74 ± 1.45	2.45 (0.50–10.30)
Total bilirubin (μmol/L)	8.51 ± 4.08	7.70 (3.20–34.80)
Hematocrit (%)	41.73 ± 4.29	41.70 (31.00–54.00)
Hemoglobin (g/L)	137.12 ± 15.73	138.00 (101.00–185.00)
Mean corpuscular hemoglobin (pg)	30.52 ± 1.58	30.50 (25.90–34.30)
Mean corpuscular hemoglobin concentration (g/L)	328.24 ± 9.32	329.00 (302.00–356.00)

**Table 2:** Drug combinations in patients with bipolar affective disorder (*n* = 99).

Drug	Category	N	Drug	Category	N
Alprazolam tablets	0	93	Metformin hydrochloride tablets	0	92
	1	6		1	7
Amlodipine besylate tablets	0	94	Metoprolol tartrate tables	0	44
	1	5		1	55
Aripiprazole tablets	0	67	Nifedipine sustained-release tablets	0	38
	1	32		1	61
Aspirin enteric-coated tablets	0	92	Omeprazole enteric-coated capsules	0	86
	1	7		1	13
Atorvastatin calcium tablets	0	91	Propranolol hydrochloride tablets	0	97
	1	8		1	2
Clonazepam tablets	0	95	Risperidone tablets	0	95
	1	4		1	4
Clozapine tablets	0	96	Silymarin capsules	0	82
	1	3		1	17
Docusate sodium tablets	0	96	Sodium valproate sustained-release tablets	0	96
	1	3		1	3
Irbesartan and hydrochlorothiazide tables	0	96	Sodium valproate tables	0	96
	1	3		1	3
Lithium carbonate sustained-release tablets	0	92	Trihexyphenidyl hydrochloride tablets	0	95
	1	7		1	4
Lithium carbonate tablets	0	94	Zopiclone tablets	0	96
	1	5		1	3
Lorazepam tablets	0	95			
	1	4			

Category: 0, without drug; 1, with drug. N is the number of patients.

### 2.2 Modeling

A quetiapine PPK model for patients with BAD was built using non-linear mixed effect modeling (NONMEM), including apparent oral clearance (CL/F), apparent volume of distribution (V/F), and the absorption rate constant (*K*_a_, fixed at 1.46/h) (23).

Equation 1 expresses the inter-individual variability:


(1) $${\text{C}}_{\text{i}}=\text{TV}\left(\text{C}\right)\times \text{exp}\left(\eta_{\text{i}}\right)$$

where *C*_i_ is the individual parameter, TV(C) represents typical individual parameter (individual parameter with representative characteristics), and *η_i_* denotes symmetrical distribution (in statistics, the distribution of index data is symmetrical; i.e., the distribution shape of the data on both sides of the mean is similar).

Equation 2 presents the random residual variability:


(2) $${\text{E}}_{\text{i}}={\text{G}}_{\text{i}}+{\text{G}}_{\text{i}}*{\text{ϵ}}_{1}+{\text{ϵ}}_{2}$$

where *E_i_* denotes the observed concentrations, *G_i_* represents the individual predicted concentrations, and *ϵ_n_* denotes symmetrical distribution.

Equation 3 displays the relationship of the pharmacokinetic parameters with weight:


(3) $${\text{L}}_{\text{i}}={\text{L}}_{\text{std}}\times {\left({\text{N}}_{\text{i}}/{\text{N}}_{\text{std}}\right)}^{\text{M}}$$

where *L_i_* is the *i*-th individual parameter, *N_i_* is the *i*-th individual weight, *N*_std_ is the standard weight of 70 kg, and *L*_std_ represents typical individual parameter. *M* is the allometric coefficient: 0.75 for CL/F and 1 for V/F (24).

Equations 4, 5 determine the pharmacokinetic parameters between the continuous covariates and the categorical covariates, respectively:


(4) $${\text{R}}_{\text{i}}=\text{TV}\left(\text{R}\right)\times {\left({\text{S}}_{\text{i}}/{\text{S}}_{\text{m}}\right)}^{\text{Z}}\;$$

(5) $$R_{i}=\text{TV}\left(\text{R}\right)\times (1+\text{Z}\times {\text{S}}_{\text{i}})$$

where *R*_i_ is the individual parameter, TV(R) represents typical individual parameter, *Z* denotes the parameter to be estimated, *S_i_* is the covariate of the *i*-th individual, and *S*_m_ is the population median for the covariate.

The stepwise method was used to evaluate the covariate, in which the objective function value (OFV) decreasing more than 3.84 (*p* < 0.05) and increasing more than 6.63 (*p* < 0.01) were the inclusion and exclusion standards, respectively (25, 26).

### 2.3 Model evaluation

The final quetiapine PPK model for patients with BAD was evaluated using visual graphics and bootstrap, where bootstrap sampling usually refers to reliable random sampling using the bootstrap framework. In fields such as data analytics and machine learning, random sampling is a common technique used to create subsets of a dataset in order to better understand and model the entire dataset.

### 2.4 Simulation

The quetiapine concentrations of patients with BAD were simulated using the Monte Carlo method, where the therapeutic window (valid therapeutic reference values) was 100–500 ng/ml (22). We simulated 1,000 virtual patients with BAD who weighed 40, 60, 80, 100, and 120 kg for 1, 4, 8, 12, 16, 20, 24, and 28 mg kg^−1^ day^−1^ quetiapine. The probability of reaching the therapeutic window was selected as the assessment index. At the same time, the present study evaluated the probability of exceeding the upper limit (maximum concentration, 500 ng/ml) of the therapeutic window, which was the safety index.

## 3 Results

### 3.1 Modeling

The final quetiapine PPK model for patients with BAD is shown as follows (Equation 6 and 7):


(6) $$\text{CL}/\text{F}=76.1\times {\left(weight/70\right)}^{0.75}$$

(7) V/F=530×(weight/70)

CL/F and V/F represent the apparent oral clearance and the apparent volume of distribution, respectively.

### 3.2 Evaluation

**Figure 1:**
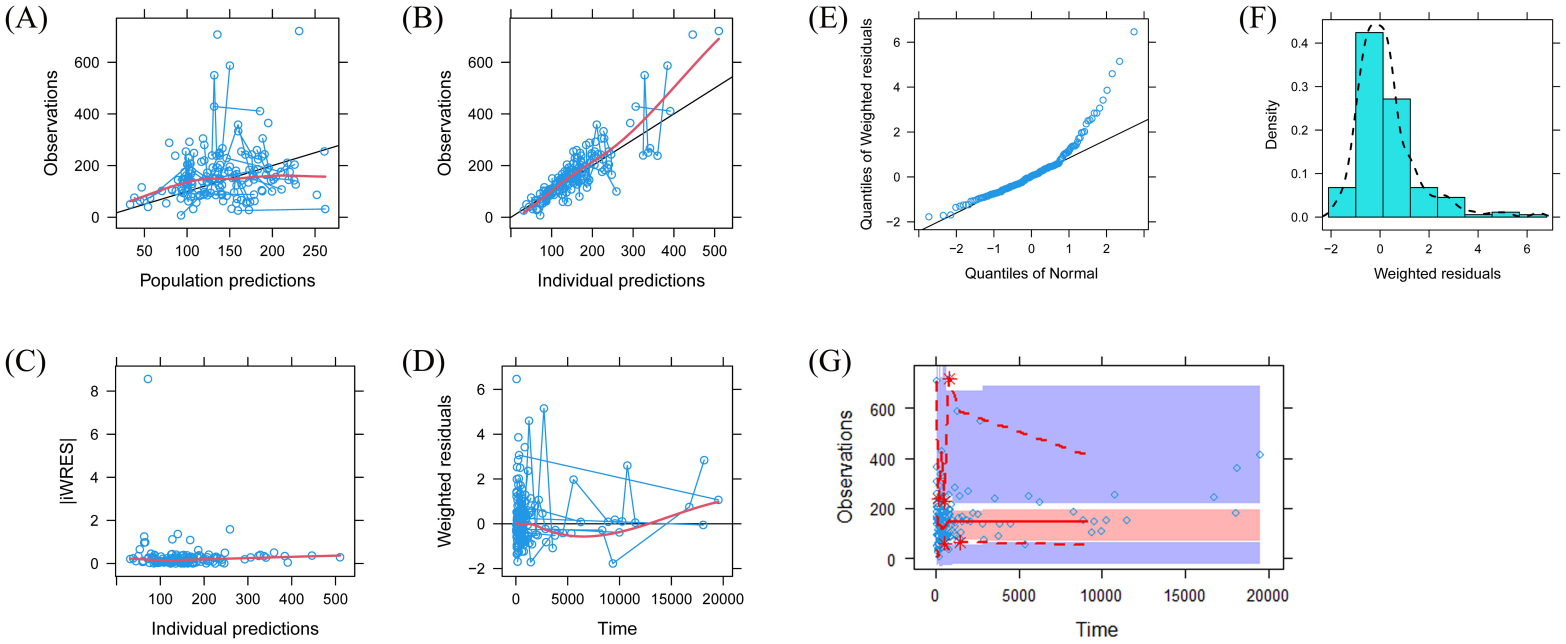
Model evaluation. **(A)** Observations vs. population predictions. **(B)** Observations vs. individual predictions. **(C)** Absolute value of the weighted residuals of individuals (│iWRES│) vs. individual predictions. **(D)** Weighted residuals vs. time. **(E)** Quantiles of the weighted residuals vs. quantiles of normal. **(F)** Density vs. weighted residuals. **(G)** Visual predictive check (VPC) of the model.

**Figure 2:**
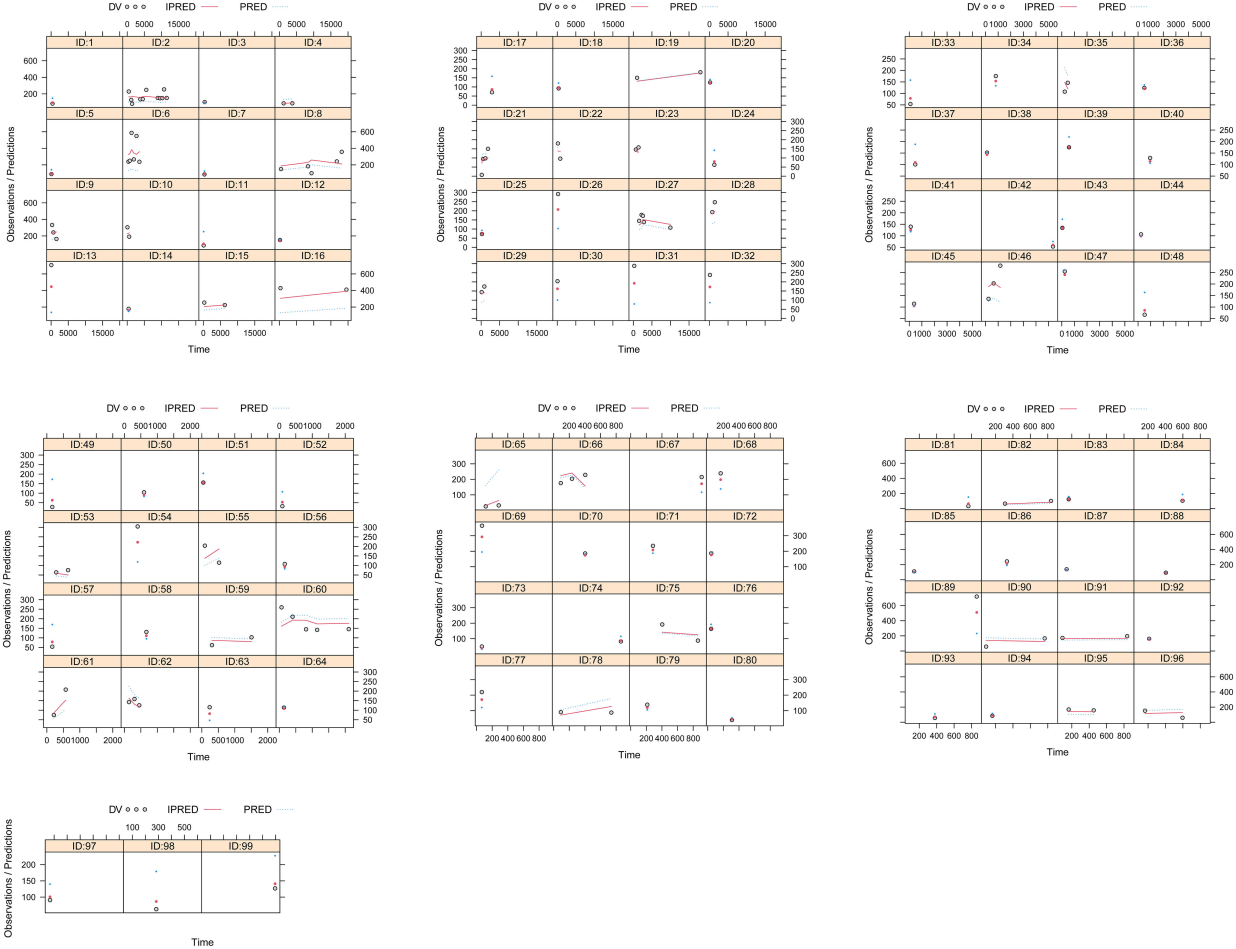
Individual plots. ID, patient ID number; DV, measured concentration value; IPRED, individual predictive value. PRED, population predictive value.

A visual representation of the quetiapine PPK model for patients with BAD is displayed in **Figure 1**, which showed that the quetiapine concentrations of these patients were well predicted by the final model. The individual plots are shown in **Figure 2**, which indicated that the quetiapine PPK model of the patients with BAD could accurately predict the quetiapine concentrations at the individual level. The results of bootstrap validation are presented in **Table 3**, which showed that the final quetiapine PPK model of the patients with BAD was accurate and reliable.

**Table 3:** Parameter estimates of the quetiapine final model and bootstrap validation in patients with bipolar affective disorder.

Parameter	Estimate	SE (%)	Bootstrap		Bias (%)
			Median	90% Confidence interval	
CL/F (L/h)	76.1	26.9	76.5	46.8–118.0	0.53
V/F (L)	530	59.6	544	210–2088	2.64
*K*_a_ (h^−1^)	1.46 (fixed)	–	–	–	–
*ω*_CL/F_	0.285	24.0	0.284	0.193–0.410	−0.35
*σ*_1_	0.312	7.9	0.310	0.262–0.348	−0.64
*σ*_2_	23.896	23.9	23.033	10.536–32.288	−3.61

The 90% confidential interval is presented as the 5th–95th percentile of the bootstrap estimates. Bias refers to the prediction error, which was calculated as: Bias = (Median − Estimate)/Estimate × 100%.

CL/F, apparent oral clearance; V/F, apparent volume of distribution; K_a_, absorption rate constant; ω_CL/F_, inter-individual variability of CL/F; σ_1_, residual variability, proportional error; σ_2_, residual variability, additive error.

### 3.3 Recommended dosage

**Figure 3:**
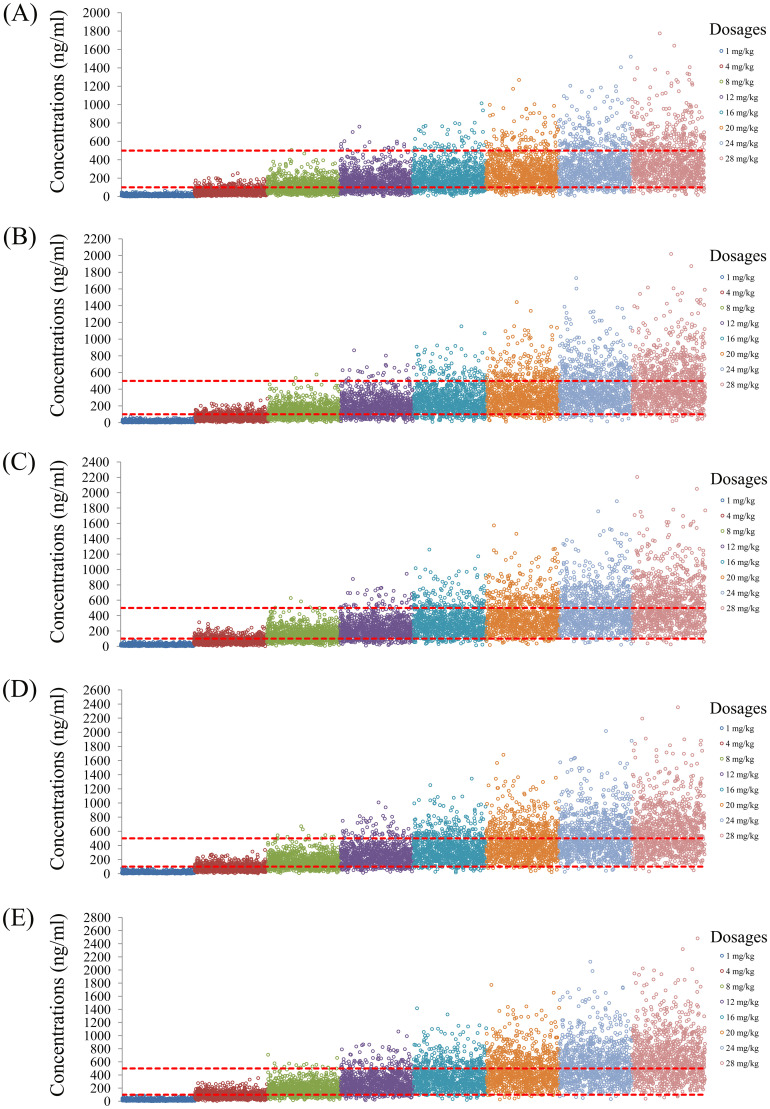
Simulated quetiapine concentrations in patients with bipolar affective disorder (BAD). **(A–E)** Patients with BAD weighing 40 kg **(A)**, 60 kg **(B)**, 80 kg **(C)**, 100 kg **(D)**, and 120 kg **(E)**.

**Figure 4:**
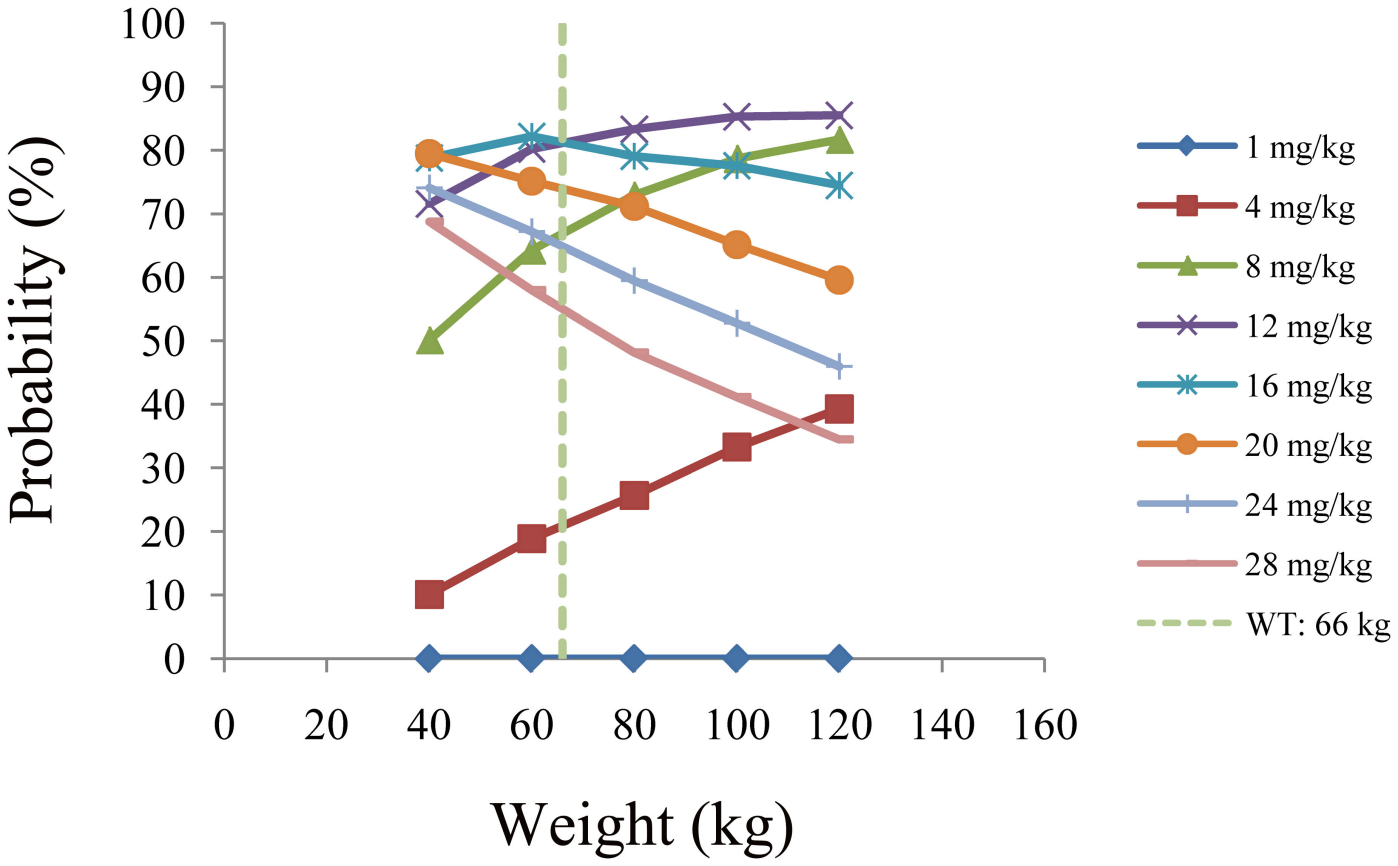
Probability of reaching the therapeutic window of quetiapine in patients with bipolar affective disorder.

The simulated quetiapine concentrations in patients with BAD are shown in **Figure 3**. **Figures 3A-E** display the data for patients with BAD weighing 40, 60, 80, 100, and 120 kg, respectively. The probability of reaching the therapeutic window of quetiapine in patients with BAD is shown in **Figure 4**. For patients with BAD weighing 40–66 kg, the initial dose recommendation was 16 mg kg^−1^ day^−1^ and the probability of reaching the therapeutic window was 78.8%–82.2%. For patients with BAD weighing 66–120 kg, the initial dose recommendation was 12 mg kg^−1^ day^−1^ and the probability of reaching the therapeutic window was 81.5%–85.5%, as shown in **Table 4**.

**Table 4:** Initial dosage recommendation of quetiapine in patients with bipolar affective disorder.

Body weight (kg)	Dosage (mg kg^−1^ day^−1^)	Probability to achieve the target concentrations (%)	Probability to exceed the upper limit of the target concentrations (%)
[40–66)	16	78.8–82.2	5.2–10.3
[66–120]	12	81.5–85.5	3.6–8.1

### 3.4 Safety evaluation

**Figure 5:**
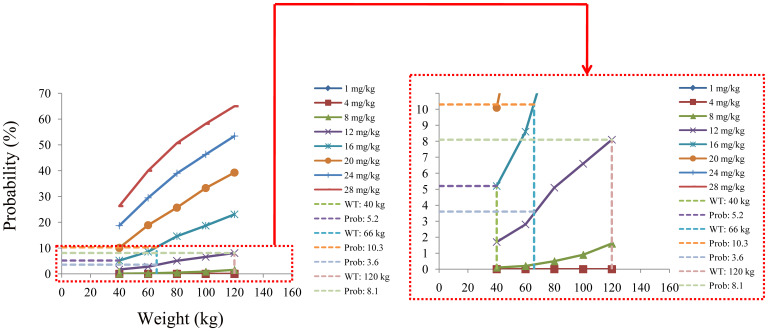
Probability of exceeding the upper limit of the therapeutic window of quetiapine in patients with bipolar affective disorder.

The probability of exceeding the upper limit of the therapeutic window (500 ng/ml) at 1,000 simulated concentrations as the safety evaluation is shown in **Figure 5**. For patients with BAD weighing 40–66 kg, the initial dose recommendation was 16 mg kg^−1^ day^−1^ and the probability of exceeding the upper limit of the therapeutic window was 5.2%–10.3%. For those weighing 66–120 kg, the initial dose recommendation was 12 mg kg^−1^ day^−1^ and the probability of exceeding the upper limit of the therapeutic window was 3.6%–8.1%, as shown in **Table 4**.

## 4 Discussion

Quetiapine is a dibenzodiazepine compound with a molecular structure similar to those of clozapine and perphenazine. It is not only effective in both positive and negative symptoms of schizophrenia but can also improve the cognitive function of patients. In addition, quetiapine has been used in the treatment of BAD (27). Quetiapine is rapidly absorbed by mouth, peaking at approximately 1.5 h and stabilizing within 48 h, with a half-life of approximately 7 h. The plasma protein binding rate is approximately 83%, while the bioavailability of oral tablets is close to 100%. In addition, quetiapine is completely metabolized *in vivo*, and its main metabolic pathway involves CYP3A4 and CYP2D6 (28). Therefore, the possible drug interactions caused by complex drug combinations in the course of clinical use deserve attention. Complex drug interactions, especially pharmacokinetic interactions, could affect the clearance rate of quetiapine and consequently affect its concentration, leading to differences in the dose requirements of different patients with BAD.

In this study, it was found that the co-medications (in tablet form, unless otherwise indicated) in patients with BAD included alprazolam, amlodipine besylate, aripiprazole, enteric-coated aspirin, atorvastatin calcium, clonazepam, clozapine, docusate sodium, irbesartan and hydrochlorothiazide, sustained-release lithium carbonate, lithium carbonate, lorazepam, metformin hydrochloride, metoprolol tartrate, sustained-release nifedipine, enteric-coated omeprazole capsules, propranolol hydrochloride, risperidone, silymarin capsules, sustained-release sodium valproate, sodium valproate, trihexyphenidyl hydrochloride, and zopiclone. To investigate whether these drug combinations and other factors affected quetiapine clearance in patients with BAD, MIPD was used.

MIPD integrated multifarious information including patients, drugs, and diseases via mathematical modeling and simulation technology, the obvious advantage of which was improving the safety, effectiveness, economy, and adherence to pharmacotherapy compared with empirical medication (29). MIPD has been widely used. For example, Zhou et al. reported on the optimization of oral isavuconazole for a population in a special physiological or pathological state using a physiologically based pharmacokinetic MIPD (35). Janssen Daalen et al. conducted a clinical trial on MIPD using machine learning for levothyroxine in general practice (30). Power-Hays et al. reported on the validation of HdxSim as a clinical decision support tool for MIPD of hydroxyurea in children with sickle cell anemia (31). Chen et al. conducted a real-world study on the initial dosage optimization of olanzapine in patients with BAD based on MIPD (32). Schatz et al. reported on the predictive performance of multi-model approaches for MIPD of piperacillin in critically ill patients (33). Villeneuve et al. reported on the rejection-free survival at 3 years of kidney transplant recipients with MIPD of mycophenolate mofetil (34). Thus, in this study, a PPK model of quetiapine was constructed for patients with BAD based on MIPD and real-world clinical data and an optimal initial dose of quetiapine for these patients was recommended.

In the final model of quetiapine in patients with BAD, the CL/F and V/F were 76.1 L/h and 530 L, respectively. The combinations of the drugs discussed above were not included into the final model affecting quetiapine clearance in patients with BAD: only body weight affected the clearance of quetiapine. Furthermore, based on Monte Carlo simulations, we predicted patients with BAD in the 40- to 120-kg weight range and found that, for those weighing 40–66 kg, the initial dose recommendation was 16 mg kg^−1^ day^−1^, the probability of reaching the therapeutic window was 78.8%–82.2%, and the probability of exceeding the upper limit of the therapeutic window was 5.2%–10.3%. For patients with BAD weighing 66–120 kg, the initial dose recommendation was 12 mg kg^−1^ day^−1^, the probability of reaching the therapeutic window was 81.5%–85.5%, and the probability of exceeding the upper limit of the therapeutic window was 3.6%–8.1%.

This study has some limitations. The influence of gene polymorphism was not considered. To the best of our knowledge, in the clinical use of quetiapine, its genotype identification is not a routine clinical test item, and the real-world data of quetiapine did not include genotype polymorphism. The MIPD model in this study was also based on real-world data to build a prediction model that can be used for real-world clinical drug use, which would more closely resemble real-world clinical use than models containing genes. In addition, the study focused on the initial dose recommendation; thus, the course of long-term treatment needs to be adjusted based on TDM in order to evaluate the efficacy and safety of quetiapine.

## 5 Conclusion

The present study, for the first time, recommended an initial dose of quetiapine in patients with BAD based on MIPD and real-world data, providing an individualized reference for the administration of quetiapine in patients with BAD.

## Data Availability

The original contributions presented in the study are included in the article/supplementary material. Further inquiries can be directed to the corresponding authors.
